# Investigating the lateralisation of experimentally induced auditory verbal hallucinations

**DOI:** 10.3389/fnins.2023.1193402

**Published:** 2023-07-06

**Authors:** Olivia Mak, Samuel Couth, Christopher J. Plack, Sonja A. Kotz, Bo Yao

**Affiliations:** ^1^Division of Human Communication, Development & Hearing, School of Health Sciences, The University of Manchester, Manchester, United Kingdom; ^2^Department of Psychology, Lancaster University, Lancaster, United Kingdom; ^3^Department of Neuropsychology and Psychopharmacology, Faculty of Psychology and Neuroscience, Maastricht University, Maastricht, Netherlands

**Keywords:** auditory verbal hallucination (AVH), signal detection, lateralisation, hearing voices, Pavlovian conditioning, neurotypical populations

## Abstract

**Introduction:**

Auditory verbal hallucinations (AVHs), or hearing non-existent voices, are a common symptom in psychosis. Recent research suggests that AVHs are also experienced by neurotypical individuals. Individuals with schizophrenia experiencing AVHs and neurotypicals who are highly prone to hallucinate both produce false positive responses in auditory signal detection. These findings suggest that voice-hearing may lie on a continuum with similar mechanisms underlying AVHs in both populations.

**Methods:**

The current study used a monaural auditory stimulus in a signal detection task to test to what extent experimentally induced verbal hallucinations are (1) left-lateralised (i.e., more likely to occur when presented to the right ear compared to the left ear due to the left-hemisphere dominance for language processing), and (2) predicted by self-reported hallucination proneness and auditory imagery tendencies. In a conditioning task, fifty neurotypical participants associated a negative word on-screen with the same word being played *via* headphones through successive simultaneous audio-visual presentations. A signal detection task followed where participants were presented with a target word on-screen and indicated whether they heard the word being played concurrently amongst white noise.

**Results:**

Results showed that Pavlovian audio-visual conditioning reliably elicited a significant number of false positives (FPs). However, FP rates, perceptual sensitivities, and response biases did not differ between either ear. They were neither predicted by hallucination proneness nor auditory imagery.

**Discussion:**

The results show that experimentally induced FPs in neurotypicals are not left-lateralised, adding further weight to the argument that lateralisation may not be a defining feature of hallucinations in clinical or non-clinical populations. The findings also support the idea that AVHs may be a continuous phenomenon that varies in severity and frequency across the population. Studying induced AVHs in neurotypicals may help identify the underlying cognitive and neural mechanisms contributing to AVHs in individuals with psychotic disorders.

## Introduction

Auditory verbal hallucinations (AVHs) are perceptions of voices in the absence of any external input. Research on AVHs has typically focused on psychiatric disorders, such as schizophrenia, as they are one of the most common positive symptoms of this disorder (Schneider, 1959; David, 1999; Baethge et al., 2005; Oorschot et al., 2012), with 70% of individuals with schizophrenia experiencing them (Turkington et al., 2019). While there remains much to be uncovered regarding the pathophysiology of AVHs, previous studies have attempted to establish how cognitive dysfunction could explain the experience of AVHs. For example, AVH severity is correlated with memory inhibition deficits in individuals with schizophrenia, as evidenced by intrusion of words recalled from previous lists in verbal memory tests (Brébion et al., 2005). Impairments in source monitoring have also been observed in this patient group, including difficulties discriminating between their own speech and that of others (Seal et al., 2004). Such findings support the inner speech theory, which proposes that AVHs arise from a misattribution of one’s own inner voice, or “inner speech,” to an external source (Frith and Done, 1989). Essentially, the person fails to recognise that they are the source of their own thoughts and thus interpret them as coming from elsewhere. However, the inner speech theory cannot fully explain the experience of AVHs (Oulis et al., 1995; Daalman et al., 2011) and much of its phenomenology remains unknown.

While AVHs are more commonly associated with psychiatric disorders, there is evidence that neurotypical individuals with no psychiatric history also experience AVHs (de Leede-Smith and Barkus, 2013; Linscott and Van Os, 2013; Moseley et al., 2014; Baumeister et al., 2017). However, there are some differences in how neurotypicals experience AVHs compared to individuals with psychiatric disorders. For example, it has been suggested that individuals with psychiatric disorders perceive AVHs as more negative in content compared to neurotypical individuals (de Boer et al., 2022). The negative emotional valence of AVH voices has even been found to accurately predict the presence of a psychiatric disorder (Daalman et al., 2011). Furthermore, a systematic review by Baumeister et al. (2017) found an earlier age of onset in non-clinical voice hearers (healthy individuals who hear voices but otherwise do not present with any clinical or psychiatric disorder), a higher perceived sense of control over the voice, and voices experienced less frequently, compared to individuals with psychiatric disorders. On the contrary, similarities have been found in AVHs between psychotic and non-psychotic groups, including the loudness of the voices, their perceived location inside or outside the head, and personification (Daalman et al., 2011).

Despite the similarities and differences in the phenomenology of AVHs, their existence in both neurotypical and psychiatric individuals lends support to the idea of a psychosis continuum (Strauss, 1969; Beer, 1996; Van Os et al., 2000; DeRosse and Karlsgodt, 2015), and that common mechanisms may underlie AVHs experiences (Diederen et al., 2012; Garrison et al., 2019). In support, Baumeister et al. (2017) found a greater prevalence of cognitive biases, reduced global functioning, and the presence of other positive symptoms such as delusions, in neurotypical individuals who experience AVHs compared to neurotypical individuals who do not experience AVHs, yet to a lesser extent compared to patient groups. In addition, the existence of schizotypal personality disorder, which can be seen as an intermediate schizophrenia spectrum phenotype, lends further credence to the notion of a psychosis continuum; schizotypal traits were found among the biological relatives of individuals with schizophrenia (Torgersen, 1985) and linked to a genetic component (Tsuang, 2000). Therefore, AVHs in neurotypical individuals can be seen as a proxy for its clinical equivalent and provide a good model for studying the cognitive mechanisms underlying AVHs in general.

To gain a better understanding of how induced hallucinations share key characteristics with clinical ones, more research exploring the experience of AVHs in neurotypical individuals is required. Studies investigating AVHs in clinical groups are often confounded by comorbidities - depression has an estimated prevalence rate of 50% in individuals with schizophrenia (Buckley et al., 2009) - medication side-effects, duration of illness, and clinical heterogeneity (Andreasen et al., 2011; Cascella et al., 2011; Arango et al., 2012; Fusar-Poli et al., 2013). Therefore, one benefit of studying neurotypical individuals is to investigate AVHs whilst controlling for these confounding factors.

### The role of expectation

One promising explanation of AVHs is that they arise from a mismatch between expectations and actual perception. Specifically, a person’s expectations may be so strong that they ‘override’ their perceptions, leading to illusory percepts (Dolgov and McBeath, 2005; Corlett et al., 2009). Such top-down influences can be seen in visual illusions, such as the Ebbinghaus illusion, whereby a central circle appears bigger or smaller depending on the size of the surrounding circles.

The powerful influence of expectations on perception can also be observed in the auditory domain through Pavlovian conditioning (Powers et al., 2017). Through frequent presentation of simultaneous auditory and visual stimuli, an association between the two can be formed such that, in conditions of white noise, presentation of the visual stimulus only may induce the detection of an auditory stimulus even when no auditory stimulus was played. This is a form of classical conditioning and induced ‘hallucinations’ (false positives; FPs) may result from this repeated pairing of the auditory and visual word presentations (Powers et al., 2017). In other words, the expectation of hearing a word can influence perception. This Pavlovian conditioning paradigm offers an avenue to investigate features of induced FPs in neurotypicals without the confounds typically reported for clinical populations.

Here, we paired the Pavlovian conditioning paradigm with a signal detection task to explore the extent to which induced FPs in signal detection share similar features to clinical AVHs. Signal detection theory (SDT), applied to decision-making under uncertainty, proposes that decisions in discriminating between ‘noise’ and ‘signal + noise’ in a typical signal detection task are based on whether each individual’s response criterion is met on any given trial. Measures of perceptual sensitivity and response bias can be derived from four possible outcomes (correct hit, correct rejection, false alarm, and miss). Perceptual sensitivity measures how effective the perceptual system is at detecting the signal, while response bias represents an individual’s response criterion for deciding whether a signal has actually been perceived or not (Green and Swets, 1966). A bias toward responding more liberally and lower perceptual sensitivity were found in both voice-hearing individuals with psychiatric disorders (Moseley et al., 2022) and healthy controls (Powers et al., 2017). There was also a positive correlation between conditioned hallucinations (i.e., FPs) and hallucination proneness in both groups, as measured by the Launay-Slade Hallucination Scale (Powers et al., 2017).

Studies reporting similar performance in SDT tasks in individuals with schizophrenia experiencing AVHs and neurotypicals who scored high on self-report measures of hallucination proneness adds further support for the continuum hypothesis. Barkus et al. (2007) demonstrated more FPs on an auditory signal detection task for neurotypical individuals with high proneness to AVHs, compared to those with medium or low proneness. Moreover, FPs activated similar brain regions (superior and middle temporal cortex) to those observed during AVHs in individuals with schizophrenia (Barkus et al., 2007). In another auditory SDT task, neurotypical individuals with high hallucination proneness also differed from those with low proneness on a measure of response bias (Bentall and Slade, 1985), which reflects the cognitive aspect of perception, i.e., the tendency towards being either conservative or liberal in one’s decision-making. This pattern of results was also found in individuals with schizophrenia with and without AVHs - the former showed more liberal responding (similar to neurotypicals highly prone to hallucinations) - while perceptual sensitivity was the same across patient groups (Bentall and Slade, 1985). Taken together, these results suggest that there may be similar mechanisms underlying AVHs in both clinical and non-clinical populations, which seem to be driven primarily by response bias.

There is also evidence that high hallucinators have higher vividness of auditory imagery than low hallucinators (Mintz and Alpert, 1972). More vivid auditory imagery has been associated with AVH severity in individuals with schizophrenia actively experiencing hallucinations (Aleman et al., 2003). Auditory imagery may be more relevant for neurotypical individuals who might not always be prone to hallucinate. Therefore, the current study also investigated whether auditory imagery predicts induced FPs.

### Using Pavlovian conditioning to explore left lateralisation of induced FPs

One feature of general language processing is the right ear advantage in dichotic listening of linguistic stimuli, which provides evidence for left-lateralised language processing (Kimura, 1961; Geffen, 1978). This is due to the superior efficiency of the contralateral neural pathway from the right ear to the speech processing areas in the left hemisphere (Kimura, 1961). Neuroimaging studies have found that this lateralised advantage may originate in the left Perisylvian region (Jung et al., 2003; Brancucci et al., 2004, 2005; Van den Noort et al., 2008). Studies reported a smaller right ear advantage for dichotic words in individuals with schizophrenia compared to individuals with depression, which was associated with positive symptoms. This advantage held true even when controlling for antipsychotic medication, which is consistent with a dysfunction in the left hemisphere (Bruder et al., 1995). This was shown to be specific for hallucinating individuals with schizophrenia (rather than positive symptoms more broadly) who showed no right ear advantage compared to non-hallucinating individuals with schizophrenia (Green et al., 1994). Similarly, in the meta-analysis of dichotic listening studies conducted by Ocklenburg et al. (2013), they found weaker left-hemisphere language lateralisation in individuals with schizophrenia compared to healthy controls, which was significantly more pronounced in those with AVHs than those without. Even when schizophrenia participants with AVHs were asked to imagine hearing a voice in one ear alone, there was no lateral bias compared to the usual right-ear advantage for healthy controls (Altamura et al., 2020). These findings indicate a reduced left-hemisphere lateralisation for language perception, which has been associated with greater AVH severity (Hugdahl et al., 2012), mediated by impaired functional and structural interhemispheric auditory connectivity (Steinmann et al., 2019). Such AVH severity, in turn, has been linked to asymmetric structural abnormalities, including reduced grey matter volume in the left insula (Romeo and Spironelli, 2022), and a smaller surface area in this area of the brain in actively hallucinating schizophrenia participants compared to healthy controls (Sone et al., 2022).

Studies using dichotic listening tasks to assess language lateralisation imply that auditory verbal hallucinations may also be left-lateralised. However, the weaker lateralisation observed in AVH-experiencing individuals with schizophrenia seems to suggest otherwise (Ocklenburg et al., 2013). To our knowledge, no studies have yet tested the lateralisation of induced AVHs using a signal detection task in neurotypical individuals. Testing this population avoids the confounds of different symptom profiles, varying attentional and cognitive deficits, and years since diagnosis found in the clinical population. It also informs our understanding of the psychosis continuum model, developing our knowledge of the extent to which the signal detection performance of the clinical and non-clinical populations differs under conditions of monaural input. To explore this further, the current study proposed presenting stimuli monaurally (left vs. right) so that FP rate could be measured for either hemisphere separately.

To summarise, this study aimed to: (1) use a Pavlovian conditioning paradigm together with a signal detection task to test the degree of left-lateralisation of FPs, perceptual sensitivity, and response bias, in neurotypical participants and (2) elucidate the relationship between FPs, sensitivity, and bias with individuals’ self-reported hallucination proneness and spontaneous use of auditory imagery.

## Methods

### Participants

Fifty participants (42 female, mean age = 23.9 (range: 18–56), SD = 7.9) were recruited through opportunity sampling. All were undergraduates, postgraduates, or staff members from the University of Manchester. All were first language English speakers, with normal hearing and no self-reported history of any psychiatric or neurological disorders. Psychology undergraduates participated in the study in exchange for course credits, responding online through a sign-up system. All other participants were compensated financially for their participation. Ten participants were excluded from the experiment due to failures in establishing their spoken word detection thresholds. Forty participants remained (34 female, mean age = 23.8 (range: 18–56), SD = 7.9).

The study was approved by the University of Manchester Research Ethics Committee in accordance with the Declaration of Helsinki 2013. All participants provided informed consent.

### Stimuli and design

A within-subjects design was used with the side of presentation as an independent variable (two levels: left ear and right ear). The spoken words used in the main experiment were ‘crazy’ and ‘stupid’, using both female and male recordings taken from Google’s Speech Synthesiser. Both were 1 s in length. These words were chosen as they have the same number of syllables and are negative.

Participants were assigned to one of four stimulus lists based on their gender and participant number. Based on their participant number, participants were then assigned to either the ‘crazy’ target word first condition, or the ‘stupid’ target word first condition. Similarly, auditory input was presented through the left ear first for half the participants and the right ear first for the other half. This was to counterbalance the lists and to control for order effects. Male participants were presented with the male recording in the experimental tasks, whilst female participants were presented with the female recording.

### Procedures

The experiment was programmed using open-source software OpenSesame (Mathôt et al., 2012), a Python cross-platform program that enables the creation of psychological experiments. Participants attended a single 30–40 min testing session in a testing cubicle in a psychology lab at The University of Manchester. They were seated in front of a computer screen in a moderately lit testing room. Before starting the experiment, participants were asked for their gender, age, and handedness, which the experimenter recorded.

### Threshold procedure

**Figure 1:**
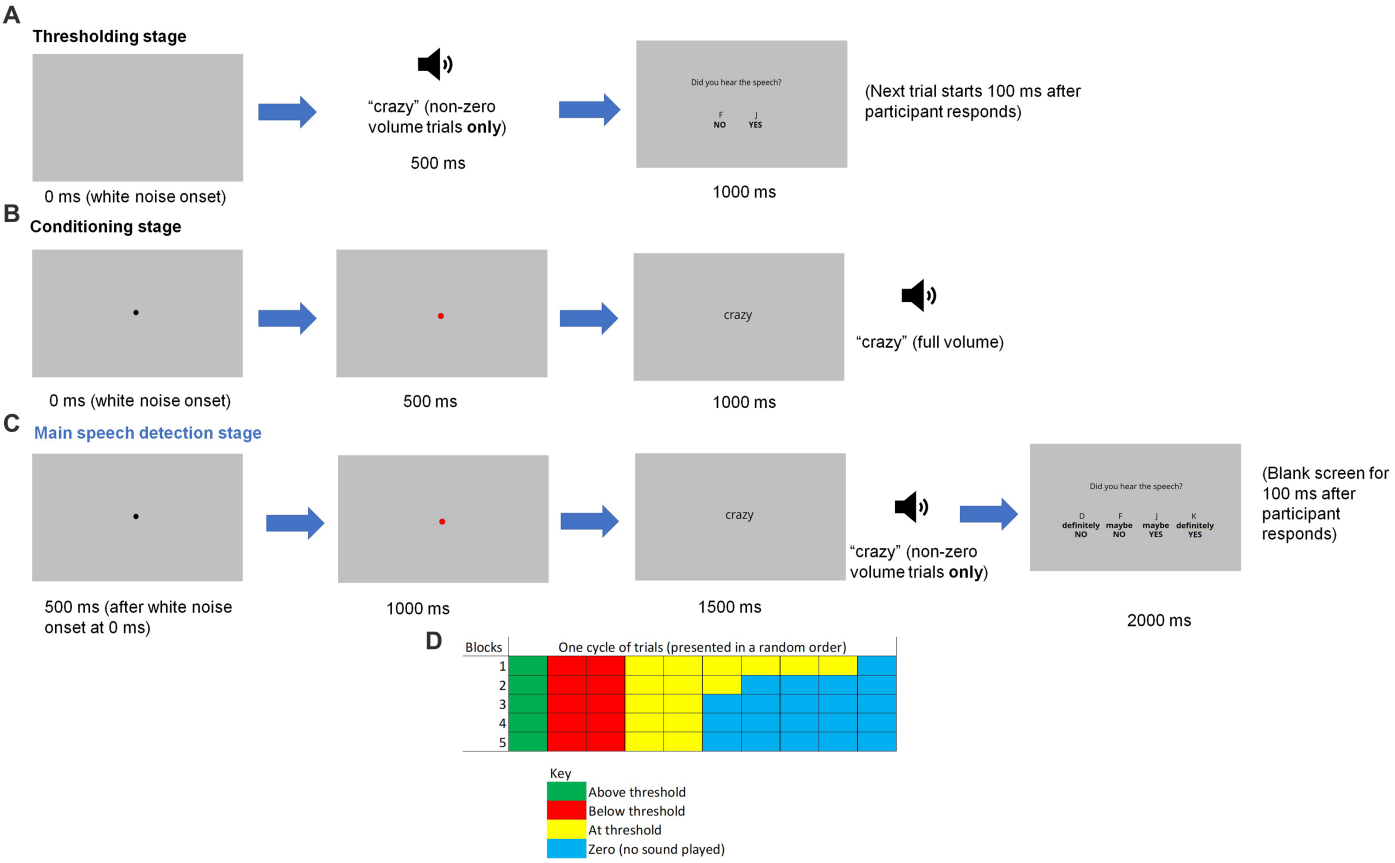
A graphical illustration of an example single trial during the **(A)** thresholding stage, **(B)** conditioning stage and **(C)** main speech detection stage, along with **(D)** a diagram showing the distribution of trial types in each block during this final stage. The loudspeaker icon represents the speech being played on non-zero volume trials.

Prior to beginning the main experiment, each participant’s individual speech detection threshold was established. Participants were asked to detect speech (i.e., ‘crazy’ or ‘stupid’) monaurally under constant white noise presented through headphones. The sound level was set at a comfortable level for participants’ hearing. Each trial began with white noise followed by the target word (‘crazy’ or ‘stupid’) after a 500 ms delay. Participants then saw the question ‘Did you hear the word?’ presented on-screen. They were asked to press the ‘F’ key for ‘no’ if they did not hear the speech and the ‘J’ key for ‘yes’ if they did (see Figure 1A). The Parameter Estimation by Sequential Testing (PEST) procedure (Taylor and Creelman, 1967) was used to obtain each participant’s speech detection threshold. In every trial, the sound level of the target speech changes dynamically depending on whether or not the participant detects the signal in white noise. The procedure ends when the presence of the target speech in white noise is perceived to be the most ambiguous, i.e., at a sound level that results in 50% detection and 50% non-detection responses. This sound level is the participant’s individual hearing threshold and is used in the main speech detection task.

Prior to the start of this thresholding phase, there were five practice trials to give the participants a sense of the different sound levels of the target speech (100% - maximum level - (twice), 60, 20 and 0% - minimum level). 100% represented the sound level of the original Google Speech Synthesiser recordings. A variety of sound levels were presented initially to familiarise the participants with the sound level changes that they would experience later in the main speech detection task. The sound level of the white noise remained constant throughout the entire experiment. The purpose of this procedure was to establish each participant’s normal hearing threshold, which allowed the variable sound level of the speech in the later speech detection task to be adapted or tailored accordingly.

### Pavlovian conditioning

Next, participants were instructed to listen to the target word (‘crazy’ or ‘stupid’), which was also presented on-screen as a visual cue, while white noise was playing simultaneously in the background. Both the word and white noise were presented monaurally. Each trial started with the white noise and a black fixation dot on the screen, which turned red after 500 ms to indicate the impending speech signal (see Figure 1B). Speech was played at 100% volume following another 500 ms, with the red dot changing to the text of the spoken word in a black font. No response was required from the participant. The next trial began 500 ms after the word was presented on screen. Each trial was presented 20 times. This number of presentations was chosen on the basis of being the optimum for paired-associative learning (Dear et al., 1967).

### Speech detection task

In the speech detection task, participants listened to white noise and saw the written word (‘crazy’ or ‘stupid’) appearing on the screen. The word was presented visually to enhance the precision of the induced expectation of hearing the word (Prat-Carrabin and Woodford, 2021). They were instructed to decide whether they could hear the target word being spoken monaurally whenever they saw the word being presented on the screen. Each trial started with the immediate onset of the white noise, with a black fixation dot appearing after 500 ms. As in the conditioning phase, this dot turned red after another 500 ms. The word appeared on the screen 500 ms later, with speech being played at the same time (except in no speech trials). Participants then saw the question ‘Did you hear the speech?’ presented on-screen, along with the following response keys: ‘D’ for ‘definitely NO’, ‘F’ for ‘maybe NO’, ‘J’ for ‘maybe YES’ and ‘K’ for ‘definitely YES’ (see Figure 1C). The letters corresponding to each response were presented on screen directly above the response statement (i.e., “D” above “Definitely No”) to remind participants which keys to press. Participants were also instructed to place their middle and index fingers of their left hand on D and F and place their middle and index fingers of their right hand on J and K, at all times to ensure an accurate response. All other response keys were disabled (pressing them elicited no response). After they pressed a relevant response key, the screen turned blank for 100 ms before the start of the next trial.

The task consisted of five blocks of trials, with 20 trials in each block. Participants took a short break between every block of trials. Speech was presented at one of four levels, which varied in a random order within a block of trials: no speech presented (defined as a ‘0’ trial), at the participant’s threshold, above the threshold (defined as the threshold x 1.5), or below the threshold (defined as the threshold x 0.5). Figure 1D shows the number of each type of trial in each block (one cell represents a single trial). There were two cycles of trials in each block. As can be seen, the number of ‘0’ trials increased in the later blocks. The purpose of gradually increasing the number of trials in which no speech was played was to build expectation; it was to develop participants’ implicit learning of the probability of ‘yes’ trials so that they may perceive speech even when none was present (i.e., a false positive), as indicated by a ‘definitely yes’ or ‘maybe yes’ response. Only the ‘0’ trials entered the final analyses.

The entire procedure (threshold, conditioning and speech detection task) was repeated for the second word (‘crazy’ or ‘stupid’, order counterbalanced between participants) with auditory input presented through the other ear.

### Questionnaires

At the end of the experiment, participants were asked to complete two questionnaires: the revised Launay-Slade Hallucination Scale (LSHSr), a 16-item questionnaire which assesses the prevalence of pathological perceptual experiences pertaining to hallucinations (Launay and Slade, 1981), and the five-item auditory imagery subscale of the Spontaneous Use of Imagery Scale (SUIS; Reisberg et al., 2003), which assesses the propensity to use mental imagery in everyday life. The LSHSr has been found to map onto five factors: sleep-related hallucinations, daydreaming, intrusive or vivid thoughts, auditory hallucinations, and visual hallucinations (Larøi and Van der Linden, 2005). It contains items such as ‘I often hear a voice speaking my thoughts aloud’ and ‘In the past, I have had the experience of hearing a person’s voice and then found that there was no one there.’ An example item of the SUIS is ‘If I need to say something important, I first imagine how I will say it in my head’.

Both questionnaires were completed on the computer. For the LSHSr, participants were told to indicate how much each statement applied to them by pressing the corresponding number keys on the keyboard (1 = certainly does not apply to me, 2 = possibly does not apply to me, 3 = unsure, 4 = possibly applies to me, and 5 = certainly applies to me). For the SUIS, they were asked to indicate how much they agreed with each statement (1 = definitely disagree, 2 = slightly disagree, 3 = neither agree nor disagree, 4 = slightly agree, 5 = definitely agree).

### Data analysis

Data were analysed using R (version 4.2.2; R Core Team, 2022). To establish the effectiveness of the conditioning paradigm, each participant’s individual threshold and FP rate was calculated by dividing the sum of their ‘yes’ responses (‘definitely yes’ and ‘maybe yes’ combined) to ‘0’ trials, where the recording was not played, by the total number of these trials. This was calculated for left and right ears separately.

For the SDT measures of perceptual sensitivity and response bias, the following was calculated for each side of presentation (left or right): the hit rate (where the participant correctly indicated they heard the word), miss rate (where the participant incorrectly indicated they did not hear the word when it has been played), and correct rejection rate (where the participant correctly indicated they did not hear the word on trials). Both perceptual sensitivity and response bias are calculated using the FP rates and hit rates. These measures are calculated, respectively, with the following formulas d’ (sensitivity) = [z(H) - z(F)] and C (bias) = − [z(H) + z(F)]/2, where z represents the z-scores of the hit and FP rates. For perceptual sensitivity, a value of 0 shows an inability to distinguish between the signal and noise while higher values indicate greater sensitivity to the signal (Stanislaw and Todorov, 1999). Response bias reflects the general tendency to respond ‘yes’ or ‘no’, with an unbiased observer having a value of 1. Values higher than 1 indicate a tendency towards more conservative responding (more ‘no’ responses) whereas those lower than 1 signify a propensity to respond more liberally (more ‘yes’ responses) (Stanislaw and Todorov, 1999).

For each measure (FP rate, perceptual sensitivity and response bias), the spread of the data was assessed to check whether they were normally distributed to see whether parametric or non-parametric tests were appropriate. The skewness for the FP distribution (averaged across both left and right ear presentation) was 0.99. A density plot confirmed that the distribution was right-skewed. The skewness for the sensitivity distribution (averaged across side of presentation) was −0.70. A density plot confirmed that the distribution was left-skewed. The skewness for the response bias distribution (averaged across side of presentation) was 0.1. A density plot confirmed that the distribution had no skew. Given that 2 out of the 3 distributions were skewed, non-parametric tests were used for all measures for consistency. All tests were rank tests because most of the measures were skewed.

To determine whether participants experienced ‘induced hallucinations’ (i.e., a significant rate of FPs), a one-sample Wilcoxon signed-rank test against a mean of 0 (collapsed across side of presentation) was conducted. A Wilcoxon signed-rank test was then conducted to see whether there was a significant difference in the FP rate between side of presentation (left vs. right). To determine whether there was a significant difference between the side of presentation (left vs. right) for perceptual sensitivity and response bias, paired-samples Wilcoxon signed rank tests between either ear were conducted, for each measure separately. The overall scores to the LSHSr and SUIS were extracted for each participant and multiple regression was conducted with these scores as predictor variables, along with side of presentation, and with FPs as the dependent variable.

To examine to what extent FPs are predicted by side of presentation, hallucination proneness and/or auditory imagery, a binomial generalised linear mixed model was fitted (estimated using ML and Nelder–Mead optimiser) with FPs as the dependent variable and side of presentation, LSHSr and SUIS as predictors. The fixed effect structure included the 3 main effects and the interactions between side of presentation with LSHS and SUIS, respectively (formula: FPs ~ side * LSHS + side * SUIS). The random effects included side of presentation as a by-subject random slope (formula: 0 + side | subject). Standardised parameters were obtained by fitting the model on a standardised version of the dataset. 95% Confidence Intervals (CIs) and value of ps were computed using a Wald z-distribution approximation. Multiple comparisons were corrected for using the Bonferroni method.

## Results

### Proportion of FPs across participants

Results showed that not all participants experienced induced FPs. Ten out of the 40 participants (25%) did not have any FPs in either ear while 9 experienced them only in the right ear (22.5%) and 7 only in the left ear (17.5%).

### FP rates and SDT measures

**Figure 2:**
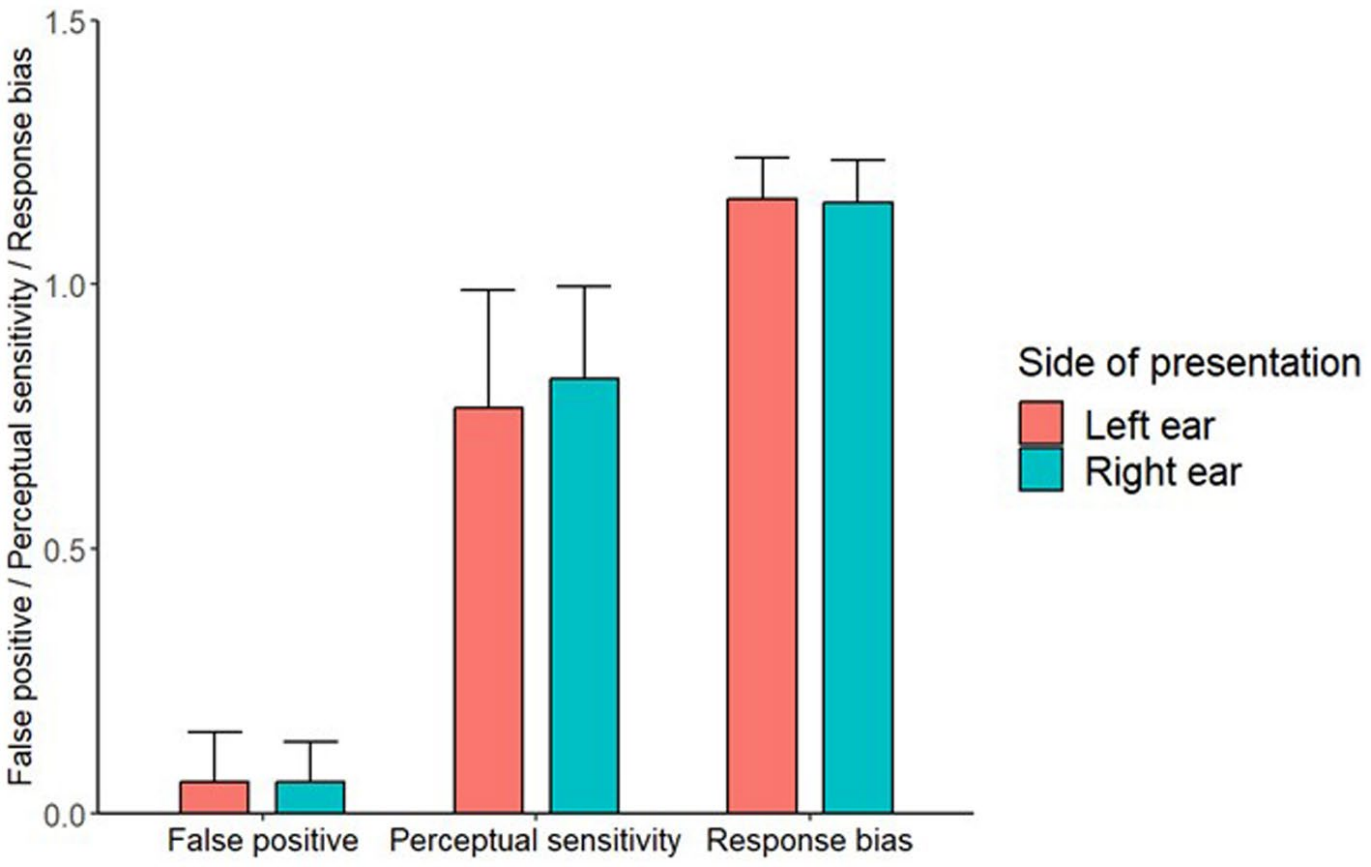
A bar chart showing the FP, perceptual sensitivity and response bias values for both side of presentation locations. Error bars represent 1 standard deviation.

The one-sample Wilcoxon signed-rank test showed that the conditioning paradigm elicited a significant number of FPs across both ears (mean = 0.059), *V* = 378, *p* < 0.001. The paired-samples Wilcoxon signed-rank test revealed no significant difference between the FP rates for the left (mean = 0.059) and right ears (mean = 0.059): *V* = 175, *p* = 0.745. The paired-samples Wilcoxon signed-rank test also showed that there was no significant difference in perceptual sensitivity between the left and right ears (*V* = 240, *p* = 0.475) (Figure 2), and no significant difference in response bias between the left and right ears (*V* = 338, *p* = 0.309) (Figure 2).

### FP rates as a function of individual hallucination proneness and auditory imagery

Total scores for the LSHSr ranged from 19 to 63 and the mean score was 36.4 (SD = 11.2). For the SUIS, total scores ranged from 13 to 25, with a mean score of 20.2 (SD = 3.2).

There was no statistically significant difference between the left and right ears in the overall model (*F*-value = 0.08, *p* = 0.735). The model’s total explanatory power was moderate (conditional *R*^2^ = 0.14), and the part of the model related to the fixed effects alone (marginal *R*^2^) was 0.04.

**Figure 3:**
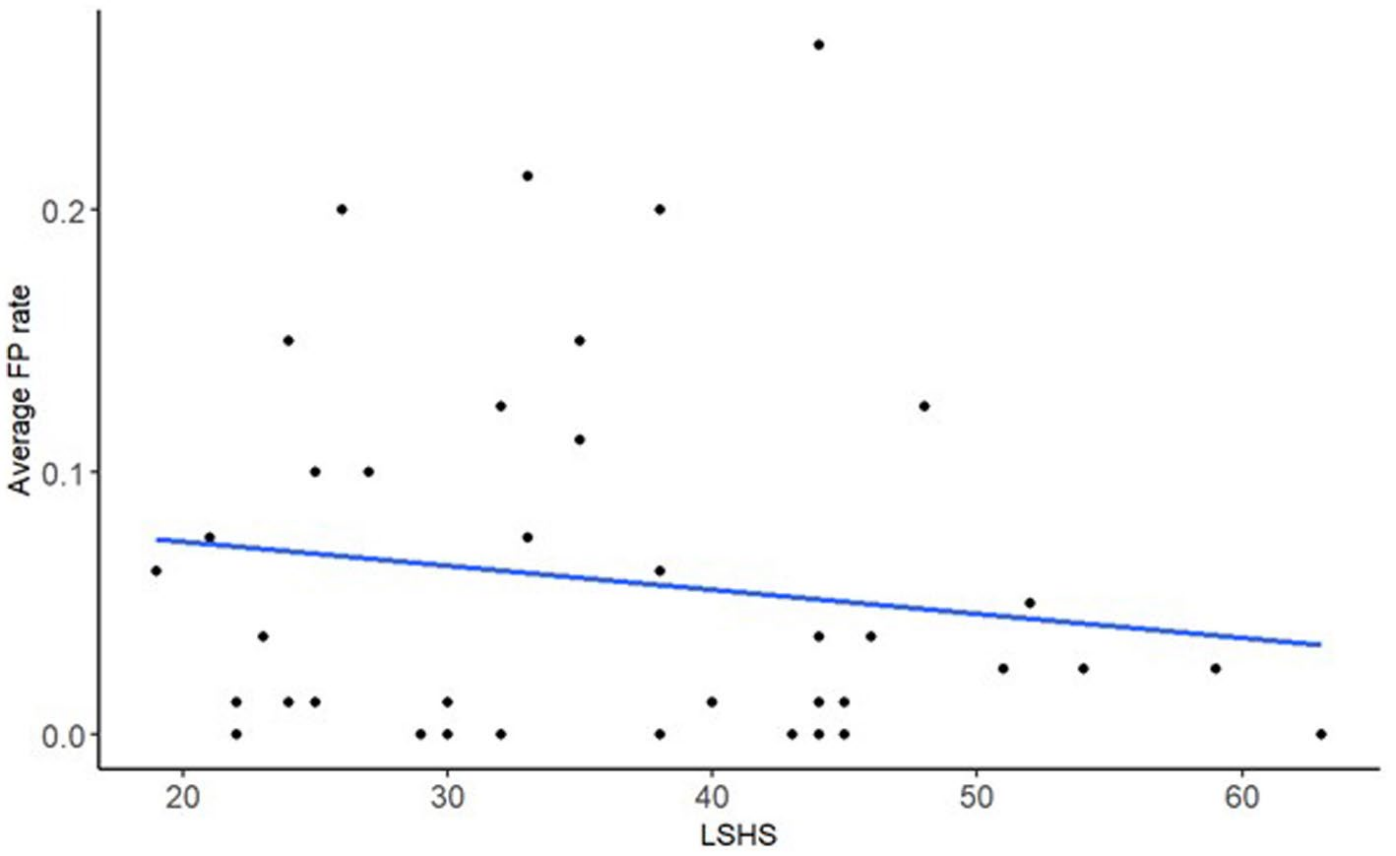
A scatter plot of the model’s significant fixed effect of FP rate on LSHS, with the regression line.

After correcting for multiple comparisons, there was a main effect of LSHS on FPs only (β = −0.49, *SE* = 0.19, *p* = 0.045), all other predictors were non-significant (Table 1). The Bayes factors of 0.57 for LSHS suggests that this effect is not robust. Figure 3 shows the negative association between LSHS and FPs.

**Table 1:** The fixed effects and interaction estimates (‘^*^’ denotes interaction), along with the standard errors, *p*-values and Bonferroni-corrected for multiple comparisons.

	*β*	SE	*z*	*p*	p_corrected_	BF_10_
Side of presentation	0.09	0.25	0.34	0.735	1.000	0.02
LSHS	−0.49	0.19	−2.60	0.009	**0.045**	0.57
SUIS	0.40	0.19	2.05	0.041	0.205	0.16
Side of presentation^*^LSHS	0.89	0.55	1.61	0.107	0.535	0.06
Side of presentation^*^SUIS	−1.35	0.56	−2.42	0.016	0.080	0.28

Any values in bold denote statistical significance. ‘p’ stands for the *p*-value while p_corrected_ is the Bonferroni-corrected equivalent. BF_10_ is the Bayes factor value and means the alternative hypothesis vs. the null hypothesis.

## Discussion

This study aimed to test whether a Pavlovian conditioning paradigm could effectively induce AVHs in signal detection in neurotypicals. The data show that it produced a reliable rate of FPs in both ears, even in people with no history of psychosis. This provides evidence that building expectation through repeated presentation of speech during earlier trials could initiate a process of implicit learning that influences speech perception in later trials. The mean FP rate of 0.06 is on par with that found in a similar sample of undergraduates in an auditory signal detection task by Alganami et al. (2017), who found a rate of 0.08 in their low hallucination proneness group (also measured using the LSHSr). Although this is lower than that found in a high proneness group (0.12) (Alganami et al., 2017), it is higher than the FP rate of 0.03 found in the neurotypical sample of Chhabra et al. (2016). While the LSHS mean for our sample (36.43) was similar to the high proneness group in Alganami et al. (2017) (38.58), they used a different experimental design. In particular, they incorporated a suggestion element, with half the trials in a ‘high suggestion’ condition where participants were told the voice would be present in 80% of the trials, and a ‘low suggestion’ one where they were told it would be present in 30% of trials (in reality, the voice was present in 60%). The FP rate in the low suggestion condition was 0.10 (and 0.14 in the high suggestion condition) but it is possible the difference with that found in our experiment may be accounted for by the higher proneness score in their sample. Chhabra et al. (2016) did not assess hallucination proneness in their neurotypical participants. However, they used a variety of words for their auditory signal detection task, which may have had the effect of diluting the FP rate due to lower frequency of exposure to each one. The current results also show no significant differences in FPs nor perceptual sensitivity and response bias between left and right ears. Neither left or right ear predicted FPs. Mean LSHS score negatively predicted FPs, suggesting that participants who are more hallucination prone were less likely to generate FPs. While there was an interaction between ear and SUIS, with auditory imagery positively predicting FPs in the right ear only, this did not survive correction for multiple comparisons. The results should be treated with caution as the Bayes factors suggest there is a lack of evidence for the alternative hypothesis for all these predictors.

The lack of left-lateralisation concords with the reduced lateralisation reported in a meta-analysis of dichotic listening studies on individuals with schizophrenia experiencing AVHs (e.g., Ocklenburg et al., 2013), suggesting that induced AVHs may share similar mechanisms with clinical AVHs. However, there have also been contradictory findings on lateralisation of clinical AVHs. In a meta-analysis of fMRI studies that investigated individuals with schizophrenia while they were actively experiencing hallucinations, Sommer et al. (2003) found significantly greater activation in language-related brain regions in the left hemisphere than those in the right. However, other studies have observed reduced lateralisation of clinical AVHs (Green et al., 1994; Hugdahl et al., 2012). One possible explanation for these discrepancies is that language lateralisation is not correlated with AVH activity *per se*, but rather the extent of negative emotional valence in AVH content (Sommer et al., 2008). For example, activation of the right inferior frontal region has been associated with production of swear words and abuse terms, usually with negative emotional content (Van Lancker and Cummings, 1999). There were also large differences between individuals with psychiatric disorders in the lateralisation of AVH activity that ranged from strong left lateralisation to strong right lateralisation, with bilateralisation also observed (Sommer et al., 2008). Hence, it is possible that lateralisation may not be a defining feature of hallucinations, whether they are experienced in people with or without psychosis.

Our results are inconsistent with research findings of left-lateralised language processing in neurotypicals in dichotic listening studies (Kimura, 1961; Geffen, 1978). The discrepancies may be explained by the nature of the auditory input and cognitive processes enlisted during the specific tasks. The right-ear advantage in dichotic listening has been found to be modulated by attention (Hiscock and Kinsbourne, 2011), with a left-ear advantage observed when participants were instructed to attend to that ear (Asbjornsen and Hugdahl, 1995; Hugdahl et al., 2009). Furthermore, behavioural accuracy appears to be associated with attention-related increases in neural activity (Tanaka et al., 2021). It has been suggested that biased processing towards the attended ear combined with inhibition of intruding input from the unattended ear is responsible for this reversal towards a left-ear advantage (Asbjornsen and Hugdahl, 1995). Evidence from neuroimaging studies support this interpretation, with enhanced neurophysiological responses in the auditory cortex contralateral to the attended ear (Alho and Vorobyev, 2007) and inhibition of the ipsilateral auditory pathway (Brancucci et al., 2004). In comparison, the current study used monaural presentation to manipulate attention. As AVHs were false perceptions of non-existent signals, there was no attentional competition between the two ears (participants only attended to one ear at a time). This may have reduced the need for dominance of one hemisphere over the other.

Irrespective of the side of presentation, our findings of a negative association between hallucination proneness and FPs go against those of Bentall and Slade (1985), who found that high-scoring hallucination prone individuals responded more liberally than low scorers, demonstrating a greater willingness to believe a signal was present. Similarly, it is interesting that auditory imagery (as assessed by SUIS) did not significantly predict FPs. The hallucination proneness finding is difficult to explain, though caution should be applied as the Bayes factor shows that the evidence for this effect remains uncertain. One possible explanation is that conditioning may have strengthened the expectation of hearing the speech across all participants, thereby eliminating individual variations in their predisposition to hallucinate the word. Such an explanation might also account for the lack of an association with imagery, which does not align with prior results that the spontaneous use of auditory verbal imagery was associated with more FPs in an online auditory signal detection task in an undergraduate sample (Segal and Fusella, 1970) and in non-clinical but highly hallucination-prone participants (Moseley et al., 2016). The former study used a harmonica chord or tone as the auditory signal while the latter used short, first-person sentences of the form ‘I am….’, followed by positively (e.g., happy) or negatively valenced (e.g., sad) adjectives. It is possible that bi-syllabic words used in our experiment may be too simplistic to engage mechanisms involved in generating imagery, whose use may only be enlisted and required in more complex listening environments. Neither of these studies used a conditioning procedure. Another potential explanation is that the conditioning in our experiment increased the likelihood of FPs but may also have reduced prior individual differences in imagery use in the process. A related possibility is that cuing participants with the word on-screen is salient enough to elicit FPs, thereby eliminating the individual tendencies to engage in auditory verbal imagery. Furthermore, both studies actively instructed participants to imagine an auditory sound (in the case of Segal and Fusella, 1970) or the sentence itself (in the first experiment of Moseley et al., 2016) prior to every signal detection trial. Even in the second experiment on the latter study, where participants were not instructed to use imagery but simply reported retrospectively how much they engaged in it, there was still a positive association with FPs. This supports the idea that even high-imagery participants in our experiment may not have used imagery much during the signal detection task.

In conclusion, Pavlovian conditioning can effectively induce FP responses in auditory signal detection in neurotypicals. These FPs, as well as the derived perceptual sensitivity and response bias, do not appear to be lateralised. The findings corroborate meta-analysis reports of reduced lateralisation in speech perception in individuals with schizophrenia who experience AVHs. Moreover, the FPs are not reliably predicted by individuals’ hallucination proneness or auditory imagery use, suggesting that conditioning may have equalised individuals’ tendencies to generate FP responses. Overall, the current study supports the idea that AVHs may be a continuous phenomenon that varies in severity and frequency across the population. Studying induced AVHs in neurotypicals may help identify the underlying cognitive and neural mechanisms contributing to AVHs in individuals with psychotic disorders.

## Data availability statement

The raw data supporting the conclusions of this article will be made available by the authors, without undue reservation.

## Ethics statement

The studies involving human participants were reviewed and approved by University Research Ethics Committee, The University of Manchester. The patients/participants provided their written informed consent to participate in this study.

## Author contributions

OM: methodology, software, formal analysis, investigation, resources, writing - original draft, writing - review and editing, visualisation, and project administration. SC: writing - review and editing, supervision. CP: writing - review and editing, and supervision. SK: conceptualisation, writing - review and editing BY: conceptualisation, methodology, validation, formal analysis, writing - review and editing, supervision. All authors contributed to the article and approved the submitted version.

## Funding

This research was funded by the Medical Research Council (MR/N013751/11) and supported by the NIHR Manchester Biomedical Research Centre (NIHR203308).

## Conflict of interest

The authors declare that the research was conducted in the absence of any commercial or financial relationships that could be construed as a potential conflict of interest.

## Publisher’s note

All claims expressed in this article are solely those of the authors and do not necessarily represent those of their affiliated organizations, or those of the publisher, the editors and the reviewers. Any product that may be evaluated in this article, or claim that may be made by its manufacturer, is not guaranteed or endorsed by the publisher.
